# Exploring TSGA10 Function: A Crosstalk or Controlling Mechanism in the Signaling Pathway of Carcinogenesis?

**DOI:** 10.3390/cancers16173044

**Published:** 2024-08-31

**Authors:** Farzad Taghizadeh-Hesary, Mobina Ghadyani, Fatah Kashanchi, Babak Behnam

**Affiliations:** 1 ENT and Head and Neck Research Center and Department, The Five Senses Health Institute, School of Medicine, Iran University of Medical Sciences, Tehran 14496-14535, Iran; 2 Chester Medical School, University of Chester, Chester CH2 1BR, UK; 3 Laboratory of Molecular Virology, George Mason University, Manassas, VA 20110, USA; 4 Avicenna Biotech Research, Germantown, MD 20871, USA

**Keywords:** cancer, cancer germline antigen, TSGA10, tumor suppressor, tumor microenvironment

## Abstract

**Simple Summary:**

This research aims to explore the role of the TSGA10 protein in cancer development, specifically in how it might influence the growth and spread of cancer cells. Scientists are particularly interested in TSGA10 because it is found in both normal reproductive tissues and cancer cells, yet seems to slow down cancer progression. The key question is why cancer cells would produce a protein that could hinder their own survival. To investigate this, the authors propose several hypotheses about how TSGA10 might be involved in carcinogenesis. They will analyze both published and unpublished studies and data to understand how TSGA10 functions at different stages of cancer. By uncovering these mechanisms, this research could lead to new targeted therapies that use TSGA10 to combat cancer more effectively, offering fresh insights and potential breakthroughs in cancer treatment.

**Abstract:**

Cancer-specific antigens have been a significant area of focus in cancer treatment since their discovery in the mid-twentieth century. Cancer germline antigens are a class of antigens specifically overexpressed in germline tissues and cancer cells. Among these, TSGA10 (testis-specific gene antigen 10) is of great interest because of its crucial impact on cancer progression. Early studies explored *TSGA10* expression in a variety of cancer types. More recent studies revealed that TSGA10 can suppress tumor progression by blocking cancer cell metabolism, angiogenesis, and metastasis. An open question regarding the TSGA10 is why cancer cells must express a protein that prevents their progression. To answer this question, we conducted a comprehensive review to engage the TSGA10 in the context of the current understanding of “malignant transformation”. This review demonstrated that TSGA10 expression level in cancer cells depends on the cancer stage across malignant transformation. In addition, we evaluated how TSGA10 expression can prevent the “cancer hallmarks”. Given this information, TSGA10 can be of great interest in developing effective targeted anti-cancer therapies.

## 1. Introduction

Cancer germline antigens (CGAs) have emerged as intriguing players in normal development and cancer progression. They are predominantly expressed in the testes, ovaries, and placenta, contributing to vital processes like spermatogenesis, yet they also make unexpected appearances in different types of cancer cells [1]. Among these, TSGA10 (testis-specific gene antigen 10) is considered due to its unique impacts on cancer phenotypes. In a nutshell, TSGA10 is an 82-kilodalton protein encoded by the *TSGA10* gene located on chromosome 2q11.2, containing at least 22 exons [2,3]. *TSGA10* coding gene was discovered by Modarressi et al. (2001) based on mRNA extraction from human testis tissue [4]. In spermatids, TSGA10 is cleaved into two fragments upon translation: a 27 kDa N-terminus fragment located in the fibrous sheath of the sperm tail and a 55 kDa C-terminus fragment located in the mid-piece of sperm [5].

Studies have explored the physiologic functions of TSGA10 in spermatogenesis [6], embryogenesis [7], and neural development [7]. However, its function in carcinogenesis is still a matter of debate. CGAs usually contribute to cancer cell proliferation, invasion, and migration [1]. However, the information regarding TSGA10 is contradictory. Some studies have introduced it as a CGA [7,8,9,10,11,12]; however, more recent studies have explored its tumor-suppressive effects. Mansouri et al. (2016) realized that TSGA10 induction could inhibit the angiogenesis and invasion of HeLa cells in vitro [13]. In line with this, Jahani et al. (2020) found that TSGA10 overexpression in a breast cancer cell line (MCF-7) can reduce their metabolic and metastatic activities [14].

In summary, some studies introduced TSGA10 as a CGA, while others found it a tumor suppressor. This duality has sparked curiosity among researchers worldwide to explore the role of TSGA10 in cancer progression. An open question regarding the TSGA10 is why cancer cells need to express a protein that prevents their progression. To answer this question, we reviewed the TSGA10 literature from the scope of “cancer hallmarks” and “malignant transformation”. The available experimental studies are primarily based on in vitro studies on cancer cell lines [13,14,15]. This issue might affect the results due to the ignorance of cancer cells as dynamic entities in living organisms, their heterogeneity, and the impacts of the surrounding tumor microenvironment (TME) [16]. This conceptual review was, therefore, conducted to re-evaluate the available literature regarding the TSGA10 in cancer progression by considering malignant transformation and cancer hallmarks.

The following two sections summarize the literature regarding the TSGA10’s role in physiologic development and tumorigenesis. Section 4 provides a synopsis of the literature pertaining to malignant transformation and cancer hallmarks. Section 5 and Section 6 provide an interpretation of the TSGA10 literature based on malignant transformation and cancer hallmarks, respectively. The last two sections present the clinical implications and conclusions of this conceptual review.

## 2. A State-of-the-Art Literature Review of TSGA10 Role in the Physiologic Development

**Figure 1:**
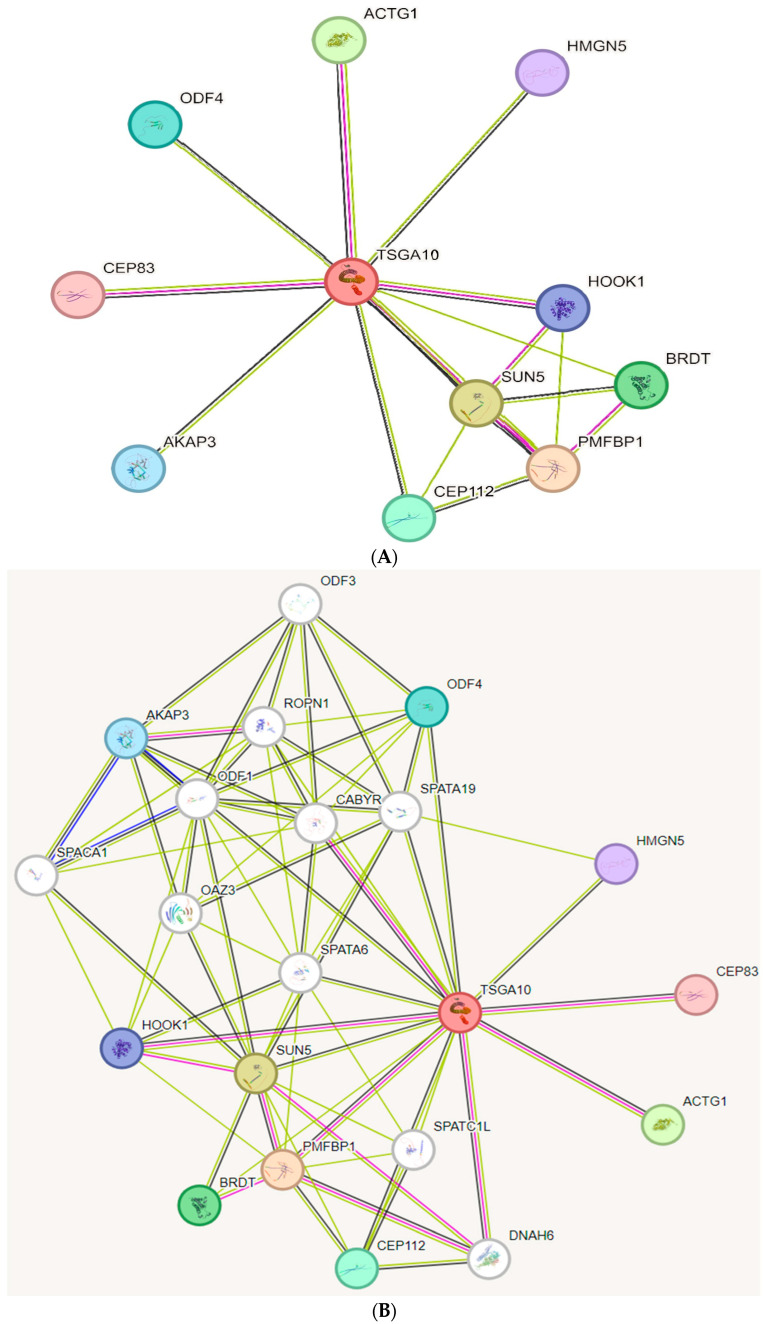
(**A**,**B**) Molecular models of TSGA10 interacting proteins and partners with other signaling pathways. Retrieved from STRING interaction network source: https://cn.string-db.org/ (accessed on 27 August 2024).

This section addresses the highlights of TSGA10 in the physiologic development. In 2001, Modarressi et al. isolated the *TSGA10* gene in the human testis and introduced its structure [4]. Upon expression, TSGA10 is spliced into two ends with distinct roles. In a mouse model, Behnam et al. (2006) demonstrated that the C-terminus of TSGA10 was implicated in the differentiation of the tail bud, small intestine, vertebrae, and the brain cortex, while the N-terminus was expressed during the development of digits [7]. Simultaneously, Aarabi et al. (2006) noted that TSGA10 expression in human testis was limited to germ cells, and lack of TSGA10 expression might negatively affect spermatogenesis and male fertility [17]. In mid-2006, Hägele et al. unveiled a crucial effect of TSGA10 by showing that TSGA10 could prevent the nuclear translocation of hypoxia-inducible factor (HIF)-1 in spermatozoa [18]. In 2010, Roghanian et al. explored that TSGA10 was expressed in dendritic cells and macrophages and interacted with vimentin through its leucine zipper motif [5]. Bioinformatic analyses have identified different proteins interacting with TSGA10 (Figure 1).

## 3. A State-of-the-Art Literature Review of TSGA10 Role in Human Malignancies

Upon introduction in 2001, Tanaka et al. (2004) realized that TSGA10 was overexpressed in a subset of melanoma (5%), colon cancer (5%), hepatocellular carcinoma (20%), ovarian cancer (35%), and prostate cancer (15%) cells [2]. Two years later, Mobasheri et al. (2006) realized that TSGA10 was expressed in 84% of patients with acute lymphoblastic leukemia (ALL) [10]. Another study demonstrated that TSGA10 was downregulated in 93% of patients with acute myeloid leukemia (AML) compared with healthy controls [19]. In an experiment on anaplastic astrocytoma cells, Behnam et al. (2009) demonstrated that C-terminus TSGA10 was located in the perinuclear region, and the N-terminus end was located in the nucleus [12]. In an in vivo study on a mouse model of esophageal squamous cell carcinoma (ESCC), Yuan et al. (2013) indicated that TSGA10 could serve as a tumor suppressor by activating the p53 or Rb signaling pathways. The investigators also demonstrated that this effect could be reversed by microRNA-577 (miR-577) binding to the 3′ untranslated regions (3′ UTRs) of TSGA10 mRNA [20]. In 2016, Mansouri et al. found that TSGA10 induction could effectively reduce the rate of angiogenesis and invasion in HeLa cells. The investigators realized that these inhibitory effects were caused by the disruption of the HIF-1 axis [13]. Asgharzadeh et al. demonstrated that the C-terminus end of TSGA10 interacted with HIF-1 with high affinity [21]. Salehipour et al. (2017) found different transcription patterns of TSGA10 in breast cancer compared with testis. This study demonstrates that TSGA10 transcripts in breast cancer cells tend to have shorter 5′ UTRs with fewer upstream open reading frames [3]. Kazerani et al. found similar findings in high-grade brain tumors compared with low-grade tumors. The authors concluded that shorter 5′ UTRs in high-grade tumors might reduce the translation efficiency of TSGA10, providing proper conditions for angiogenesis and metastasis [22]. In 2018, Bao et al. demonstrated that miR-23a-containing exosomes secreted from nasopharyngeal carcinoma cells induced angiogenesis by directly targeting the TSGA10 [23]. In line with this finding, Zhang et al. (2019) found that HIF-1 enhanced the proliferation, invasion, and migration of ESCC cells by targeting TSGA10 in a miR-10b-3p-dependent manner [24]. Hoseinkhani et al. (2019) demonstrated the negative correlation of TSGA10 with HIF-1 and VEGF (vascular endothelial growth factor) expression in patients with AML [19]. In early 2020, Jahani et al. found that TSGA10 overexpression in breast cancer cells could reduce cell proliferation and induce the G_2_/M cell cycle arrest. In addition, TSGA10 induction could reduce the cancer cells’ metabolism and metastatic ability [14]. Table 1 summarizes the key studies exploring the role of TSGA10 in different malignancies.

The following section provides a synopsis of malignant transformation and cancer hallmarks.

## 4. Stepwise Cancer Progression and Cancer Hallmarks

**Figure 2:**
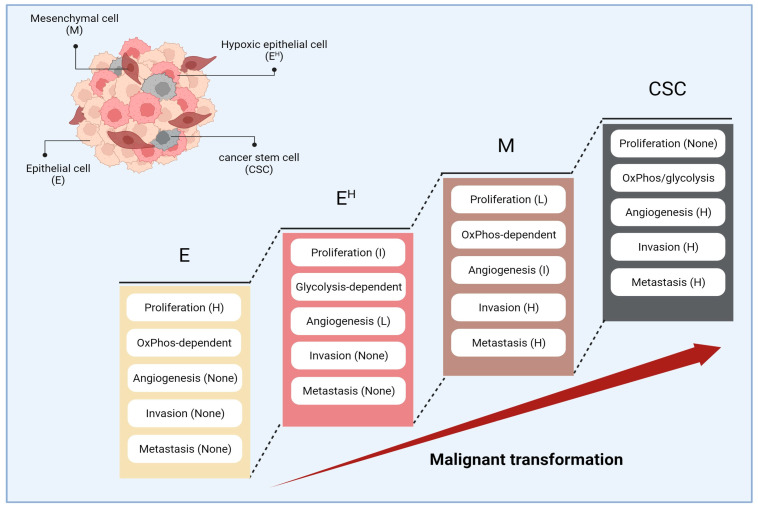
Phenotypes and capabilities of cancer cells across malignant transformation, including early-epithelial phase (E), epithelial phase at hypoxic conditions (E^H^), mesenchymal transition (M), and obtaining stemness (CSC). CSC indicates cancer stem cells; E, epithelial cancer cells at normoxia; E^H^, epithelial cancer cells at hypoxia; H, high-level; I, intermediate-level; L, low-level; M, mesenchymal cancer cells.

Extensive research on cancer biology revealed that cancer cells are not static but are dynamic, acquiring new phenotypes and capabilities during progression following sustained randomized (but programmed) changes in their genotype [29]. This process is called “malignant transformation” that makes the tumor mass “heterogenous”, containing cancer cells at different phases of malignancy with different phenotypes as well as different levels of resistance to anti-cancer treatments [30,31]. It has been demonstrated that cancer cells, after development, traverse a multistep journey, obtaining different characteristics named “cancer hallmarks” [29]. With advances in our understanding of cancer biology, the cancer hallmarks have evolved from six items in 2000 (including sustained proliferative signals, evading growth suppressors, resisting cell death, active tissue invasion and metastasis, sustained angiogenesis, and enabling replicative immortality) to ten hallmarks in 2011 (plus genome instability, tumor-promoting inflammation, deregulating cellular metabolism, and immune escape) [32,33]. In 2021, Prof. Douglas Hanahan put forward another four hallmarks, including non-mutational epigenetic reprogramming, unlocking phenotypic plasticity, senescence, and the influence of polymorphic microbes, to better illustrate the cancer phenotypes [29]. Figure 2 illustrates a summary of changes in cancer phenotype across the malignant transformation pathway.

In the initial stage of malignant transformation (proliferative phase), cancer cells try to overcome growth suppressors to secure a sustained replication. In this phase, cancer cells benefit from normoxia and sufficient micronutrients for continuous proliferation. In the proliferative phase, cancer cells’ metabolism primarily relies on oxidative phosphorylation (OxPhos) to provide the building blocks essential for replication, including amino acids, fatty acids, and nucleosides [34].

Following the increase in number of cancer cells and disturbance of the supply-demand balance, cancer cells obtain new features enabling them to survive and progress in the hypoxic, hypoglycemic, and acidic TME. In this stage, the cancer cells’ metabolism primarily depends on glycolysis. Even in this hard-to-survive condition, cancer cells can continue proliferating using glycolysis intermediates to generate the macromolecules essential for cell division [33]. The phenotypic transformation of cancer cells in this phase is conducted by several transcription factors, including HIF-1 [35]. Under normoxia, HIF-1 is ubiquitinated, following hydroxylation by prolyl hydroxylase domain (PHD) proteins and activation of von Hippel Lindau protein (pVHL) [36]. This process does not happen in hypoxic conditions, and the intact HIF-1 can conduct the transcription of numerous mediators supporting cancer cells to survive and progress in the harsh TME [37].

With the advances in tumor growth, certain cancer cells enter a dedicated transition pathway to obtain mesenchymal phenotype, a process called epithelial–mesenchymal transition (EMT). During EMT, epithelial cancer cells lose their intercellular connections and proliferative ability and obtain mesenchymal phenotypes with enhanced invasive and migratory abilities [38]. In this stage, the cancer cells’ metabolism depends on OxPhos [38]. EMT is primarily regulated by several dedicated transcription factors, including Snail, Twist, and ZEB. It has been demonstrated that ZEB and Twist were transcribed by HIF-1 [39,40], and Snail required HIF-1 for its stability [41]; therefore, EMT is an HIF-dependent process.

Following progression, a set of cancer cells obtain “stemness” phenotypes. These cells are called cancer stem cells (CSCs). The source of CSCs is the topic of debate and expanding research. Some believe they develop from normal stem cells or their progenitors (a.k.a. transit-amplifying cells) following successive oncogenic mutations [42], while others know their origins in differentiated cells [43]. An evolving idea considers EMT as the driving force of stemness. Proponents of this concept believe that EMT converts cancer cells in a terminally differentiated state to a metastable state, providing an opportunity to express new genes and, thereby, obtain new phenotypes [44]. In support, it has been demonstrated that the induction of EMT transcription factors (Snail, Twist, and ZEB) has enhanced the expression of stem cell markers (e.g., CD44) and their tumor sphere-forming ability (reviewed in [44]). CSCs’ metabolism relies on both OxPhos and glycolysis. It has been demonstrated that CSCs could switch their metabolism to glycolysis in hypoxia and OxPhos in normoxia [45].

This section illustrated the synopsis of the literature on the multistep progression of cancer. In each step, the cancer cell has a specific genetic signature replying to its metabolism and objectives. The next section discusses the role of TSGA10 in cancer progression based on the current understanding of malignant transformation.

## 5. Interpretation of TSGA10 Studies Based on the Malignant Transformation

**Figure 3:**
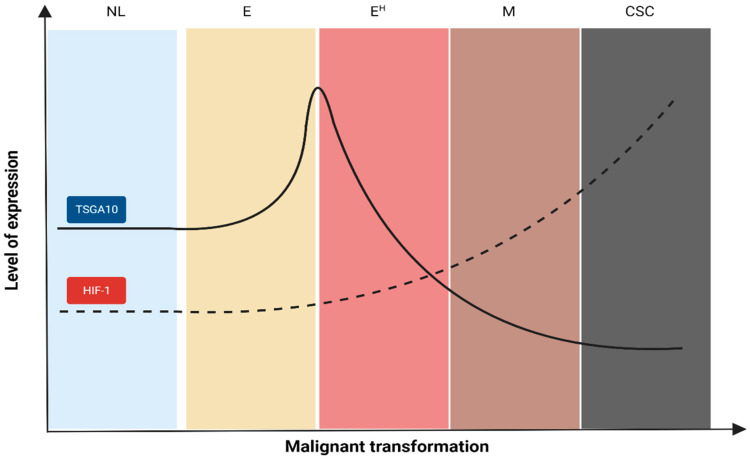
Changes in the TSGA10 and HIF-1 expression levels across the malignant transformation. CSC indicates cancer stem cells; E, epithelial cancer cells at normoxia; E^H^, epithelial cancer cells at hypoxia; M, mesenchymal cancer cells; NL, normal (noncancerous) cells.

Based on the current knowledge of the malignant transformation, our interpretation of TSGA10 in cancer progression is as follows:

In vivo studies demonstrated wide-range expression levels of TSGA10 in cancer cells (Figure 2 of [2] and Figure 1 of [19]). This finding might be due to the “heterogeneity” of cancer cells in the tumor mass with different phases of malignant transformation. In support, Kazerani et al. found a higher expression of TSGA10 in low-grade brain tumors compared with high-grade tumors [22]. Another study demonstrated a similar pattern in ESCC cells. Yuan et al. realized that TSGA10 expression significantly decreased with tumor grade, primary tumor size, and clinical stage [20]. Figure 3 illustrates our assumption on trends in TSGA10 expression during distinct steps of malignant transformation and its association with HIF-1 expression. Given the stepwise progression of cancers [46], we propose the following scenario to explain the changes in the TSGA10 level.

At the early phase of cancer initiation (proliferative phase), cancer cells primarily tend to proliferate to generate the tumor mass. Considering the importance of TSGA10 in centrosome assembly [47], it can be concluded that cancer cells upregulate the TSGA10 expression to respond to this endpoint (the increasing trend in Figure 3). However, in the following, cancer cells need to downregulate TSGA10 to respond to their progression. Cancer cells require a number of tools to progress, even in the hypoxic TME. One of these advanced tools is HIF-1, a transcription factor that improves the cancer cells’ survival, progression, and resistance in multiple ways [48]. It has been demonstrated that TSGA10 has counter-regulatory effects with HIF-1. In human sperms, the C-terminal domain of TSGA10 prevents the nuclear localization of HIF-1 during spermatogenesis [18]. This counteraction has also been demonstrated in human cancers. Jahani et al. (2020) found that TSGA10 overexpression in breast cancer cells could reduce the expression of HIF-1 target genes, including MMP7 (matrix metalloproteinase-7), GLUT1 (glucose transporter 1), CXCR4 (C-X-C chemokine receptor type 4), CXCL12 (C-X-C motif chemokine 12), LOXL2 (lysyl oxidase-like 2), and vimentin [14]. These inhibitory effects would disrupt the cancer cells’ metabolism (by reducing GLUT1) and their invasion and migratory abilities (by reducing MMP7, CXCR4, LOXL2, CXCL12, and vimentin). LOXL2 is a catalytic enzyme that cleaves the collagen cross-linking. It has been demonstrated that LOXL2 was an essential mediator of angiogenesis [49]. Therefore, the inhibitory effect of TSGA10 on LOXL2 expression can potentially inhibit angiogenesis, a point not mentioned in Jahani et al.’s article. Meanwhile, Amoorahim et al. (2020) demonstrated that TSGA10 overexpression in human umbilical vein endothelial cells (HUVECs) could inhibit endothelial cell proliferation and migration, thereby, angiogenesis, by disrupting the HIF-2α axis [50]. Jahani’s and Amoorahim’s studies demonstrate that TSGA10 overexpression can inhibit cancer cell metastasis by reducing its migratory ability and disrupting angiogenesis.

It has been demonstrated that HIF-1 has inhibitory effects on TSGA10 in cancer cells. An in vitro study on ESCC cells demonstrated that HIF-1 overexpression could downregulate TSGA10 expression by inducing miR-10b-3p expression. This study indicated that miR-10b-3p overexpression could improve cancer cell invasion and metastasis in a mouse xenograft model [24]. Another study demonstrated that miR-577 could directly regulate TSGA10 expression in ESCC cells by binding to the 3′UTR of the *TSGA10* gene. In this study, Yuan et al. found that miR-577 overexpression effectively boosted cancer cell proliferation and enhanced the transition from the G_1_ to S phase by downregulating TSGA10 expression [20]. These pieces of information illustrate that cancer cells need to decrease the TSGA10 expression in the advanced stages of malignant transformation to increase their ability to metastasize (the decreasing trend in Figure 3).

## 6. Interpretation of TSGA10 Studies Based on the Cancer Hallmarks

This section outlines how TSGA10 can prevent distinct cancer hallmarks:

**Enabling replicative immortality** and **resisting cell death:** When cells undergo malignant transformation, they employ a unique mechanism to evade replicative senescence and subsequent cell death. This involves elongating their telomeres using telomerase, allowing them to continue proliferation and avoid cell death [51]. It has been demonstrated that telomerase activity in cancer cells is HIF1-dependent [52]. Recent evidence in breast cancer stem cells shows that HIF-1 is essential for NANOG-mediated telomerase reverse transcriptase (TERT) gene expression [53]. As noted, TSGA10 prevents the HIF-1 axis [13,14]. Therefore, cancer cells must downregulate TSGA10 to have a continuous replication;

**Genomic instability:** Under physiologic conditions, it is imperative to synchronize centriole duplication with DNA replication to guarantee that each daughter cell obtains only one pair of centrioles. However, cancer cells develop “centrosome amplification”, which is the aberration in centrosome shape, size, position, and number. This condition increases the chance of aneuploidy and genomic instability across the cell divisions [54]. The physiologic assembly of centromeres relies on a set of regulators, including centrosomal protein 135 (CEP135), which is a centriole assembly protein [55]. It has been demonstrated that CEP135 was dysregulated in some breast cancers [56]. Carvalho-Santos et al. demonstrated that TSGA10 interacted with CEP135 and contributed to the physiologic assembly of centriole and basal body [57]. Therefore, downregulation of TSGA10 during cancer progression can increase the chance of genomic instability and provide less differentiated cancer cells, favoring cancer progression;

**Deregulating cellular metabolism:** The metabolism of cancer cells varies from normal cells. Cancer cells can run glycolysis even in the presence of enough oxygen pressure, a process known as “aerobic glycolysis”. This characteristic enables cancer cells to survive even in the harsh conditions of TME, like hypoxia, acidosis, and low-glucose levels [58]. It has been demonstrated that mitochondria are the main regulators of cancer cell metabolism [59]. The available literature regarding the association between TSGA10 and mitochondria is limited. Luo et al. (2020) demonstrated that TSGA10 expression is essential to organize the mitochondria along the midpiece of sperm [60]. Similar to this effect can also be speculated in cancer cells, considering the following two assumptions:

(a) Mitochondria trafficking is an essential component in malignant transformation. It has been demonstrated that cancer cells with high affinity to metastasis had fragmented mitochondria in their periphery, likely to provide enough energy for invasion. However, mitochondria in cancer cells with less metastatic affinity are mainly located in the perinuclear region in the fused form [61];
(b) Jahani et al. demonstrated that TSGA10 induction in MCF-7 breast cancer cells decreased ROS production [14]. Given that mitochondria are the main source of ROS in cancer cells [62], Jahani et al.’s finding can reflect the decrease in mitochondrial metabolism following TSGA10 activation.

This finding, in addition to the perinuclear localization of C-terminus TSGA10 [12], put forward a concept that TSGA10 overexpression can translocate the mitochondria to the perinuclear region, facilitating their fusion and reducing their metabolic activity. This concept needs to be examined in future experimental studies;

**Inducing or accessing vasculature:** Cancer cells require access to oxygen and micronutrients to have a sustained proliferation. This access is achieved by releasing angiogenic factors (e.g., VEGF) and the breakdown of extracellular matrix using MMPs [63]. As noted, TSGA10 has a negative correlation with VEGF [19]. Furthermore, Asgharzadeh et al. demonstrated that the interaction between TSGA10 and HIF-1 can modulate the epidermal growth factor receptor (EGFR) [21]. Given the importance of EGFR in the angiogenesis process [64], TSGA10 may influence angiogenesis through its interaction with EGFR. In addition, Jahani et al. demonstrated that TSGA10 could reduce the expression of MMP-7 in cancer cells [14]. Therefore, a decrease in cellular TSGA10 level is required for angiogenesis and vascular access;

**Activating invasion and metastasis:** Cancer metastasis is in two forms: single-cell and collective. Each type of metastasis requires epithelial cells to be transformed into the mesenchymal counterpart through EMT [63]. It has been well established that EMT is a HIF-dependent process [65]. Therefore, cancer cells must downregulate their TSGA10 level to obtain mesenchymal phenotype;

**Avoiding immune destruction:** Cancer cells exploit different mechanisms to shield from immunosurveillance, such as reducing antigen presentation, expressing immune checkpoints, and converting the condition of surrounding TME too harsh for immune cell recruitment (e.g., acidity) [59]. Among these, the expression of CGAs has been put forward as an immune escape mechanism. Kortleve et al. (2022) evaluated the association between the expression level of fifteen CGAs (including TSGA10) and immune escape in a pan-cancer model. Their study demonstrated that TSGA10 expression negatively correlated with the tumor-infiltrating lymphocytes and MHC molecules [66]. This study also indicated the negative correlation between TSGA10 and cancer-associated fibroblast (CAF) infiltration [66]. With reduction in TSGA10 level, the rates of CAFs in TME is elevated, which provides several benefits for cancer cells promotion by providing substrates for OxPhos of cancer cells (including pyruvate, lactate, and glutamate) [67], transferring mitochondria to cancer cells via nanotubes to support their metabolism [68], inducing tumor-promoting autophagy by releasing β-HB, IGF1/2, and CXCL12 [69], and inhibiting anti-tumor immune response [70]. The causative association between TSGA10 and the immune microenvironment needs to be addressed in immune-competent models and assessment of immunophenotypes and immunokinetics;

**Tumor-promoting inflammation:** TME consists of noncancerous cells modified to support the cancer cells’ survival, progression, and treatment resistance. The major cellular components of TME are cancer-associated fibroblasts (CAFs), myeloid-derived suppressor cells (MDSCs), tumor-associated macrophages (TAMs), tumor-associated neutrophils (TANs), and regulatory T cells (Tregs) [71]. As noted earlier, a decrease in TSGA10 expression (in advanced phases) is correlated with CAF infiltration into TME [66]. It has been demonstrated that CAFs can induce the recruitment of tumor-promoting immune cells (TAMs, MDSCs, TANs, and Tregs) toward TME in several mechanisms [70]. Therefore, a decrease in TSGA10 expression in advanced phases of malignant transformation can contribute to developing tumor-promoting inflammation. Co-culture and organoid models could help dissect the TME-specific effects of TSGA10.

## 7. The Potential Clinical Implications of TSGA10 Upregulation in Cancer Cells

**Mesenchymal–epithelial transition:** Given the close association between TSGA10 and centrosomes [47], the involvement of centrosomes in cellular polarity (as a feature of epithelial cells versus mesenchymal cells), and a reduction in invasion capabilities of cancer cells with TSGA10 overexpression, one may conclude that TSGA10 may serve as a running factor of the mesenchymal–epithelial transition (MET). In support, it has been demonstrated that TSGA10 overexpression in MCF-7 breast cancer cells upregulated the expression of E-cadherin [14], a classical biomarker of epithelial cells [72]. In addition, Jahani et al. found that TSGA10 upregulation led to the downregulation of vimentin, a classic biomarker of mesenchymal cells [14]. It has been established that vimentin was one of the main drivers of EMT, by which cancer cells obtain special phenotypes to invade the extracellular matrix and withstand the external sheer forces during metastasis [63].

Supporting evidence is the interaction between centrosomal CEP135 and TSGA10. CEP135 plays a crucial role in centrosome organization, a process essential for maintaining cellular polarity [73]. Carvalho-Santos et al. demonstrated that TSGA10 interacted with CEP135 and likely contributed to the physiologic assembly of centriole and basal body [57]. With this information, one may conclude that TSGA10 upregulation can contribute to the cancer cells to retrieve their polarity. It has also been demonstrated that more organized centrosomes could prevent aneuploidy and genomic instability [74]. Therefore, upregulation of TSGA10 might prevent the cancer cells from being promoted to the less differentiated phenotypes. Considering the importance of EMT in stemness [44] and the high resistance of CSCs to radiotherapy [75], chemotherapy [76], and immunotherapy [77], TSGA10 induction can serve as a potential modality to reduce treatment resistance.

**Exosome secretion:** Tumor progression is significantly influenced by the secretion of extracellular vesicles, which are produced in larger quantities by cancer cells compared to normal cells. These vesicles, including exosomes, carry biomolecules such as microRNAs (miRNAs) that inhibit multiple target genes and alter intercellular communication, promoting metastasis [78]. Based on the available evidence, TSGA10 can interact with several proteins involved in exosome formation and secretion, including actin [5], Rab27 [47], HIF-1 [14], and ODF2 [47]. TSGA10 interacts with actin-rich structures, influencing exosome release and connecting to Rab27 proteins that regulate exosome secretion. Its overexpression may induce apoptosis and autophagy by affecting exosome dynamics. Additionally, TSGA10 interacts with HIF-1a and p53 [13,20], potentially promoting exosome release in cancer cells. These interactions suggest that TSGA10 plays a significant role in cancer progression through exosome regulation, although further research is needed to fully understand its mechanisms.

**Radiotherapy:** This modality is the mainstay of cancer treatments and is extensively applied to different malignancies [79,80]. The main cytotoxic effect of radiotherapy is targeting the vital macromolecules of cancer cells, especially DNA [81]. It has been demonstrated that the G_1_ and S phases of the cell cycle are more radioresistant, and the G_2_ and M phases are more radiosensitive [82]. As alluded to above, TSGA10 induction can lead to G_2_/M arrest in cancer cells [14]. This effect can serve as a potential approach to improve the radiosensitivity of cancer cells. In addition, it has been demonstrated that TSGA10 overexpression in endothelial cells could induce cell cycle arrest [50]. Ironically, tumor neovascularization enhances hypoxia by forming immature, leaky vessels undergoing collapse in the extracellular matrix with high interstitial fluid pressure [83]. Therefore, TSGA10 overexpression in tumor endothelial cells can enhance the tumor oxygen pressure that inherently improves radiosensitivity [84]. The potential effects of TSGA10 induction on radiosensitivity are speculative and need testing in appropriate preclinical models.

**Immunotherapy:** It has been demonstrated that HIF-1 expression improved the cancer cells’ ability to evade the immune system [85]. The HIF-1 axis suppresses the innate and adaptive immune response by inducing the secretion of immunosuppressive factors (prostaglandin E_2_ and transforming growth factor-β) [86], expression of programmed death protein-ligand 1 (PD-L1) on cancer cells [87], and reducing tumor-associated antigen presentation via major histocompatibility complex class I (MHC-1) [88]. In addition, HIF-1 signaling can induce MDSC accumulation in TME [89], which inhibits the immune response in several ways, including by attracting M2 macrophages and Tregs into TME, impairing lymphocyte adhesion to endothelial cells and expression of immune checkpoint molecules (PD-L1) [90]. Given the inhibitory effects of TSGA10 on the HIF-1 axis, TSGA10 overexpression can provide an opportunity to enhance the anti-tumor immune response and, thereby, response to immunotherapies. The potential impact of TSGA10 induction on immunotherapy requires evaluation in suitable experimental studies.

**Anti-mitochondrial therapy:** Emerging evidence on mitochondrial metabolism has been put forward as a determining factor in cancer biology and treatment resistance [59]. This concept has recently evolved into introducing mitochondrial metabolism as a new aspect of personalized cancer treatment [91]. A big hurdle in targeted anti-mitochondrial therapies is that mitochondria are present in all human cells, and anti-mitochondrial therapies can harm normal cells. This issue limits the application of broad-spectrum anti-mitochondrial agents. As noted earlier, TSGA10 might target the mitochondrial metabolism and trafficking in cancer cells. The specific expression of TSGA10 in cancer cells can provide an opportunity to limit the anti-mitochondrial effects on cancer cells. This concept can be engaged to design targeted therapies to limit mitochondrial metabolism, specifically in cancer cells.

Future works are suggested to address the following objectives: (a) to perform multi-omics and lineage tracing to better characterize TSGA10’s context-specific functions; (b) to develop robust in vitro and in vivo models manipulating TSGA10 to validate proposed mechanisms; (c) to use unbiased screening to identify TSGA10 interactome and downstream effectors; (d) to evaluate combination therapies engaging TSGA10 along with standard care in clinically relevant models; and (e) to address key questions around therapeutic index, delivery challenges, biomarkers, and resistance for clinical translation.

## 8. Conclusions

The available TSGA10 literature poses a big question, “Why do cancer cells need to express a protein that prevents their progression?”. This review outlines that TSGA10 expression level in cancer cells depends on the cancer stage across malignant transformation. It demonstrates that in early phases, cancer cells overexpress TSGA10 to respond to their endpoint to proliferate; however, as cancer cells progress to the advanced phases of malignancy, the level of TSGA10 is reduced to allow HIF-1 to take the wheel of cancer cells and conduct the EMT and metastasis. In other words, we assume that TSGA10 is a “basic tool” for the early progression of cancer. Once cancer cells plan to enter the advanced phases, they improve their equipment to more “advanced tools” to lose their polarity, develop genomic instability, improve their mitochondrial metabolism, undergo EMT, and recruit more CAFs into TME to support their progression. The various tumor-suppressive effects of TSGA10 on cancer biology can provide a potential opportunity to enhance the efficacy of different cancer therapies. The mechanisms proposed may not prove causation. Further experimental studies are needed to explore the role of TSGA10 in cancer progression. In addition, engaging TSGA10 induction as a therapeutic approach assumes it can be safely upregulated in cancer cells without adverse effects, which requires investigation.

## Figures and Tables

**Table 1:** Comparative TSGA10 alteration in different cancers.

Cancer Types	Discussed Mechanisms	TSGA10 over Expression	TSGA10 Downregulation
Esophageal Squamous Cell Carcinoma [20,24]	TSGA10 acts as a tumor suppressor as it inhibits tumor growth by regulating the cell cycle and inducing apoptosis. Typically, downregulated in more advanced stages, larger and poorly differentiated ESCC, which leads to increased cell proliferation and malignancy.	Can it help regulate tumorigenesis?	MiR-577 functions as an oncomir as it promotes cancer progression by targeting and downregulating TSGA10. Under hypoxic conditions, the expression of miR-10b-3p would be enhanced, therefore targeting TSGA10 and reducing its expression.
Primary cutaneous T-cell lymphoma (CTCL) [25]	TSGA10 acts as a tumor-associated antigen and a candidate for targeted immunotherapy in primary CTCL and suggests a role in the immune response against tumor cells.	TSGA10 is overexpressed as a potential tumor-associated antigen in primary CTCL.	Likely reduce the immune system’s ability to recognize and target the cancer cells; hence, less effective immune surveillance and, potentially, cancer progression.
Breast Cancer [3,14,21,26,27]	A paradoxical relationship is observed between TSGA10 expression and cellular migration. The high-affinity interaction of TSGA10 C-terminal domain with HIF-1α affects 8 key proteins (VEGFA, HSP90AA1, AKT1, ARNT, TP53, VHL, JUN, and EGFR) in cancer progression.	TSGA10 overexpression is associated with reduced metastasis. TSGA10 overexpression decreases metastatic and metabolic activities, thereby reducing cell proliferation and metastasis.	TSGA10 is typically downregulated in breast cancer, which leads to cancer progression and metastasis.
Brain Tumor [12,22]	Unknown. TSGA10 is specifically expressed in astrocytes.	TSGA10 is overexpressed in brain tumors.	TSGA10 Downregulation may disrupt normal cell cycle control, which could lead to decreased cell proliferation.
Nasopharyngeal Carcinoma [23]	miR-23a regulated angiogenesis by directly targeting TSGA10. Metastasis-associated miR-23a from NPC-derived exosomes plays an important role in mediating angiogenesis by targeting TSGA10.	Overexpression of TSGA10 can counteract the effects of miR-23a and result in inhibiting proliferation, angiogenesis, and cell migration and invasion.	Suppression of TSGA10 is associated with tumorigenesis via enhancing the migration of endothelial cells, suggesting that angiogenesis is regulated by miR-23a as it directly targets TSGA10 and represses its antiangiogenic functions.
Hepatocellular Carcinoma (HCC) [28]	TSGA10 acts as an immunogenic protein that can elicit an immune response; hence, TSGA10 plays a significant role in the progression and prognosis of hepatocellular carcinoma.	TSGA10’s overexpression is linked to tumor aggressiveness, poor patient outcomes, and serves as a potential immunogenic target.	Downregulation of TSGA10 is associated with increased cell proliferation and reduced apoptosis.
AML/ALL [10,19]	*TSGA10* acts as a tumor suppressor gene in AML, as it negatively regulates the expression of VEGF by interacting with HIF-1α. TSGA10 may be involved in the proliferation of leukemic cells.	TSGA10 Overexpression leads to VEGF and HIF-1α downregulation, consequently inhibiting tumor growth and angiogenesis. TSGA10 is overexpressed in ALL, leading to proliferation of leukemic cells.	Decreased expression of TSGA10 in AML leads to increased VEGF and HIF-1α levels, promoting tumor growth and angiogenesis.
Pan-cancer studies - Melanoma - HCC - Colon - Ovarian - Prostate [2,11]	Irregular expression of TSGA10 in various cancers can affect the proliferation of cancer cells, suggesting its role in tumorigenesis.	TSGA10 is overexpressed in a subset of melanoma (5%), colon cancer (5%), HCC (20%), ovarian cancer (35%), and prostate cancer (15%), leading to increased cell division and growth, altered apoptosis, enhanced cell migration and invasion, and activation of oncogenic pathways.	Downregulation of TSGA10 can lead to a potential tumor suppression.
